# A survey in Mexico about ethics dumping in clinical research

**DOI:** 10.1186/s12910-019-0378-6

**Published:** 2019-06-03

**Authors:** Novoa-Heckel Germán, Bernabe Rosemarie

**Affiliations:** 1 National Autonomous University of Mexico (UNAM), University Bioethics Program, Mexico City, Mexico; 2 Institute of Health and Society, Faculty of Medicine, University of Oslo, Oslo, Norway

## Abstract

**Background:**

The exportation of unethical practices to low- and middle-income countries (“Ethics Dumping”) has been conceived as a prevalent practice which needs to be examined more closely. Such a practice might point towards the exploitation of vulnerable population groups. We conducted a survey among Mexican research ethics committee members to explore the issue of ethics dumping in Mexico by understanding how its existence and contributing factors and norms are perceived by these ethics committee members.

**Method:**

We designed an exploratory survey based on a five-point Likert scale, following an established, validated and published methodology. The questionnaire included both open close-ended questions. The aspects covered in the questionnaire were introductory questions on the existence of ethical issues; general perception on ethics dumping in Mexico; lack of voluntariness, undue inducement, and therapeutic misconception as exploitation risks; existence of exploitative practices; norms facilitating ethics dumping; acceptable levels of benefit to Mexico; boundaries of ethics dumping. The survey was administered to a sample of research ethics committee members from public and private Mexican hospitals in 2016.

**Results:**

Most of the ethics committee members believed clinical trials are generally ethically sound, though almost a majority think that ethics dumping is a common occurrence and that it is a serious issue. Most agree that ethics dumping needs to be addressed. They also identified other issues such as ethical issues related to patient participation and ethics committees. Further, most committee members agree that undue inducement and therapeutic misconception affect voluntariness, and that both individuals and communities receive appropriate benefits.

**Conclusion:**

From the perspective of Mexican research ethics committee members, ethics dumping commonly exists in Mexican clinical trials, as well as several related issues such as ethical issues on patient participation and ethics committees, as well as voluntariness issues. Further, most members believed these issues need to be addressed. However, most were also of the opinion that clinical trials are generally ethically compliant. This points to the need for further studies on the reasons for these perspectives.

**Electronic supplementary material:**

The online version of this article (10.1186/s12910-019-0378-6) contains supplementary material, which is available to authorized users.

## Introduction

The subject of ethics dumping has been discussed within the European literature and in our article in the journal *Bioética y Derecho* as the ethically complex situation in which clinical trials from sponsors from high income countries (HICs) are conducted in low and middle-income countries (LMICs) while exporting practices that are not acceptable in the sponsor’s country [1–3] . This ties in with acknowledged issues of exploitation and vulnerability between the HICs and LMICs [4, 5]. The EU TRUST project, for example, places “exploitation in North-South collaborations” [6] at the essence of ethics dumping, with exploitation referring to a wrongful act because of the disadvantage it causes on the vulnerable [6, 7]. The project then itemized generic exploitation risks to persons, institutions, local communities, countries, animals, and the environment [6]. We shall allocate the term “exploitation risks” in this manuscript to refer to research practices that could disadvantage the vulnerable.

Exploitation and vulnerability are, of course, sensitive topics for professionals who carry out research in Mexico. In order to explore their opinions about ethical dimensions in clinical research, we decided to conduct a survey among research ethics professionals, specifically, members of Mexican Research Ethics Committees (M-RECs) about the presence of ethics dumping and the related “exploitation risks” [6] of lack of voluntariness, undue inducement, therapeutic misconception, poor benefit-risk balance, and perceived participant responsibility to cooperate in research. *Specifically, the objective of this study is to explore the issue of ethics dumping in Mexico by understanding how its existence and contributing factors and norms are perceived by ethics committee members.*

By advancing this objective, we wish to contribute to the ongoing discussion in the literature on unethical practices in international clinical trials by providing the Mexican context. The Mexican context is an interesting case as Mexico is one of the high-middle income countries in Latin America with the most number of patients in pivotal clinical trials submitted to the European Medicines Agency for marketing authorization applications [8]. Mexico is also part of the region which is second in terms of average annual growth of clinical trials [9]. By looking at the Mexican case, we hope to contribute to the various contexts and faces of ethics dumping.

## Methods

This is an exploratory survey on the perception of M-REC members on the issue of ethics dumping in Mexico, specifically on the latter’s existence and contributing factors. To be able to invite as many M-REC members as possible given time and fund constraints, this study used an electronic questionnaire with both open and close-ended questions.

The questionnaire was created through a consultation with an expert group composed of seven individuals (a psychologist, colleagues from the Philosophy department, and members of the Ethics committee association) to formulate a series of themes and questions to explore the experiences, perceptions, and opinions of M-REC members on the ethical aspects of clinical research related to ethics dumping. (See Additional file 1 for final set of questions). With this expert group, we decided on the following themes for the questionnaire: introductory questions on the existence of ethical issues; general perception on ethics dumping in Mexico; lack of voluntariness, undue inducement, and therapeutic misconception as exploitation risks; existence of exploitative practices; norms facilitating ethics dumping; acceptable levels of benefit to Mexico; boundaries of ethics dumping. The resulting series of closed and open-ended questions addressed our research interests in a Likert scale format, as described in a previously published paper [10] . The Likert scale allows for five responses: strongly disagree, disagree, neither agree nor disagree, agree, and strongly agree. A short pilot testing of the draft questionnaire to five of the colleagues of the first author was performed.

A list of M-REC members was secured from the National Research Ethics Committees Association (www.amcei.org). Among the members in the list, members from states with active clinical trials were chosen. These M-REC members were professionals of diverse specializations with at least one-year experience with clinical research. The survey questionnaire, along with a letter of explanation, were sent to 90 members from 18 M-RECs (it should be stated that the government body that registers ethics committees (Conbioética) stated that there are currently 190 committees around the country). An Ethics Committee consulted waived the need for approval of the protocol on grounds that no risks would be involved, and that the legislation did not require approval for this type of investigation. Nevertheless, In the letter of explanation, participants were asked if they would voluntarily like to participate, leaving open the possibility not to collaborate. Of the 90, 77 responded. After the final selection where some questionnaires were dropped due to incomplete answers, 62 questionnaires were included. The electronic questionnaire was sent on 26 July 2016 and the last questionnaire was received on 22 September 2016.

The responses were systematized and integrated using an electronic medium. Quantitative data were entered and scored using Excel, as we have done in previous researches. Excel was also used to obtain the corresponding summaries and analyses. Descriptive statistics was used to summarize demographic data (average, mean, minimum and maximum values, percentages) and for frequency analysis. Qualitative data were organized, tabulated, and categorized using MS Word. Conclusions were drawn from statistical analysis, the manifest content of the answers (for open-ended questions), and through contextual interpretations.

The data obtained were coded and safeguarded in keeping with the Documentation Best Practices guidelines [11]. The anonymity of the participants was kept. Because of the documentary and anonymous nature of the survey, the approval of an ethics committee was not needed in Mexico.

## Results

Table 1 shows the demographic data of the population surveyed. The demographic variables are as follows: age, gender, years of experience in research, time devoted to research (average, range, and standard deviation (S.D.)), level of education, state of origin and type of REC affiliations.

**Table 1:** Demographic data

Demographic Data		Average	Range	S.D.	N	%
Age						
Women (years)		49	27–35	±11.8	27	43.5
Men (years)		54	23–78	±13.5	35	56.5
Total		51	23–78	±12.9	62	100
Experience in clinical research (Avg. years)		12	1–50	±11.5	62	100
Time devoted to clinical research (%)		33	0–100	±0,3	62	100
Education (n)	Undergraduate degree				11	17.7
	Medical specialization				28	45.1
	Master’s degree				15	24.1
	Doctorate				8	12.9
	Total				62	100
State of origin (n)	Mexico City				25	40.3
	Jalisco				7	11.2
	Puebla				6	9.6
	Guanajuato				3	4.8
	Nuevo León				3	4.8
	Aguascalientes				2	3.2
	Edo de México				2	3.2
	Oaxaca				2	3.2
	San Luis Potosí				2	3.2
	Veracruz				2	3.2
	Baja California				1	1.6
	Chiapas				1	1.6
	Colima				1	1.6
	Michoacán				1	1.6
	Morelos				1	1.6
	Sinaloa				1	1.6
	Yucatán				1	1.6
	Zacatecas				1	1.6
	Total				62	100
Type of institution REC (n)	Private				28	45.1
	Public				14	22.5
	N/A				20	32.2
	Total				62	100

### Introduction to the topic and general exploration of ethics issues

**Fig. 1:**
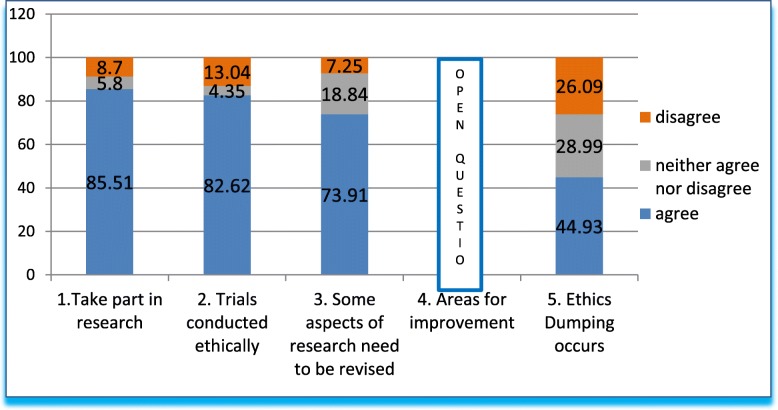
Answers to questions 1–5 in grouped percentages

The first five questions, Q1-Q5, (Fig. 1, Table 2) were designed to introduce the topic and explore ethics issues in general. In Q1 we inquired about participation in clinical research, to which 85% of respondents answered in the affirmative. The average for experience in research was 12 years, with 33% of professional activity being devoted to research (see Table 1). Less than 10% reported no participation, while 5% neither affirms nor denies it. In Q2, we stated that clinical research in the country was ethical. Over 80% of respondents agreed, whereas 13% disagreed; almost 4% neither agreed nor disagreed. Q3 sought to insist, by asking whether some ethical aspects of research should be revised. Almost 75% of respondents agreed, while 7% disagreed and nearly 20% were unsure. Q4 was an open-ended question summarized in Table 2 (“Areas that should be revised”).

**Table 2:** Survey question 4 (Areas that need improvement) and the participants’ comments

Broad categories	Replies
Patients (15 mentions)	Conditions, rights, safety, follow-up, autonomy, actual volunteering, compensation, selection, management, care, vulnerability, adverse events.
Committees (12 mentions).	Reexamining ongoing research, member profile, requirements, policies, auditing, member training and education, CONBIOÉTICA monitoring, numbers, researchers, certification, protocol revision times.
Consent (8 mentions).	Informed consent letters
Regulations (7 mentions).	Knowledge, regulation processes, best practices, applications, ethics.
Industry (5 mentions).	Dependence, bioethics and bioethics education for management, coercion, corruption, agreements, conflicts of interest.
Researchers (5 mentions).	Experience, ethics, planning and selection, ethic education, thought leaders.
Protocols (4 mentions).	Truthfulness, exclusion criteria, diagnostic criteria, treatment design, patient selection, patient follow-up.
Sites (4 mentions).	Certifications, requirements, screening by authorities, proceedings and operation.
Declaration of Helsinki (4 mentions).	Providing medication at the end of the study, giving patients information, benefits for individuals and the community.
Others (3 mentions).	Management and operation of health services [*Authorities*]. Intervention by specialists in philosophy of science [*Justification in clinical research*]. Data protection. [*Ethics in a study evaluation, monitoring*].

Q5 introduces the subject of “ethics dumping” with a brief definition. When asked whether this practice occurred in our country, nearly 50% of respondents agreed.

Q4 asked respondents to suggest areas that need improvement; the summarized answers are shown in Table 2, which presents the answers to the first open ended question. Participants may contribute more than once. The categories with the greatest number of responses were the following: patients, committees, and consent.

### General perception of ethics dumping in Mexico

**Fig. 2:**
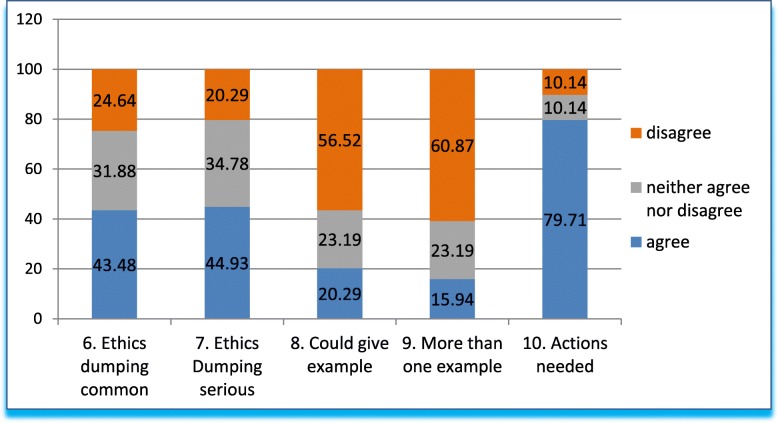
Answers to questions 6–10 in grouped percentages

Q6 to Q10 (Fig. 2) explore the general perception of the participants on ethics dumping in Mexico. Q6 elaborates on the topic of ethics dumping, asking whether this practice is common in our country. The answers are like those obtained in the previous question, with nearly 50% of respondents expressing agreement. Q7 asks about the severity and urgency of the situation. Again, the answers are similar: nearly 50% agree that the situation is serious and urgent; close to 20% do not believe this to be the case, and a third of respondents are unsure. Q8 elicits related examples, and in this case only 20% says they could provide an example of ethics dumping. When probed for knowledge of more than one example, in question 9, only 16% of respondents stated that they could provide more than one example. It is worth noting that close to 2/3 of respondents do not have multiple examples at hand. Finally, as to whether actions should be undertaken to combat the phenomenon (question 10), over 75% of respondents agreed; only 10% disagreed, and the remaining 10% had no opinion.

### Exploitation risks: lack of voluntariness, undue inducement, and therapeutic misconception

**Fig. 3:**
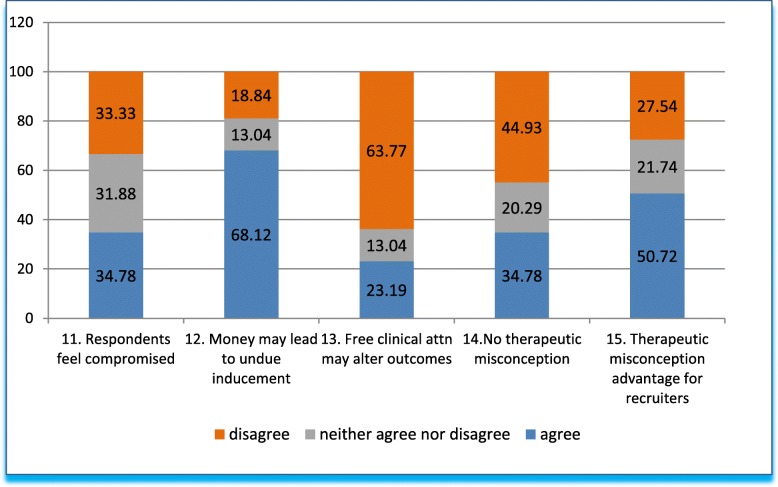
Answers to questions 11–15 in grouped percentages

Q11 to Q15 (Fig. 3) explore lack of voluntariness, undue inducement, and therapeutic misconception on a potentially vulnerable population, i.e., commonly identified contributing factors (also termed as “exploitation risks”, as explained above) to ethics dumping. This set of questions started by asking about the level of freedom that the participants themselves felt they had in responding to the questionnaire. In Q11, we analyzed whether respondents felt free to answer openly and honestly. We asked if they felt compromised in discussing these issues. Respondents agreed, disagreed or neither agreed nor disagreed in equal proportions: 33% in round figures for each of the three options.

Q12-Q13 explores the topic of undue inducement, i.e., an ethically problematic situation in research when inducements are too much that rational decision making in terms of trial participation is compromised. Q12 asks whether the promise of free medical attention and money for transportation unduly influences the decision to enroll in a trial. Q13 asks whether offering free medical attention could alter the study outcomes. As to the question of whether the decision to enroll is influenced by the promise of free medical attention, 2/3 of respondents agreed that this is true. In response to the follow-up question, 2/3 do not believe that free medical attention influences study outcomes.

Q14, with regard to therapeutic misconception, i.e., the misconception that the purpose of a clinical trial is therapy (that inclusion of a patient in a trial is primarily decided by therapeutic (as opposed to study) considerations), a third of respondents think that patient-participants are aware that the primary purpose of a trial is research (and not their health), almost half were of the opinion that patient-participants think that their participation is primary for therapeutic purposes, while nearly 20% remained undecided. Further exploring this subject, Q15 asks whether the persistence of therapeutic misconception promotes enrollment and represents an advantage for recruiters. Approximately half of respondents believe this is so, close to a third disagreed, and a little over 20% were undecided.

### On exploitation

**Fig. 4:**
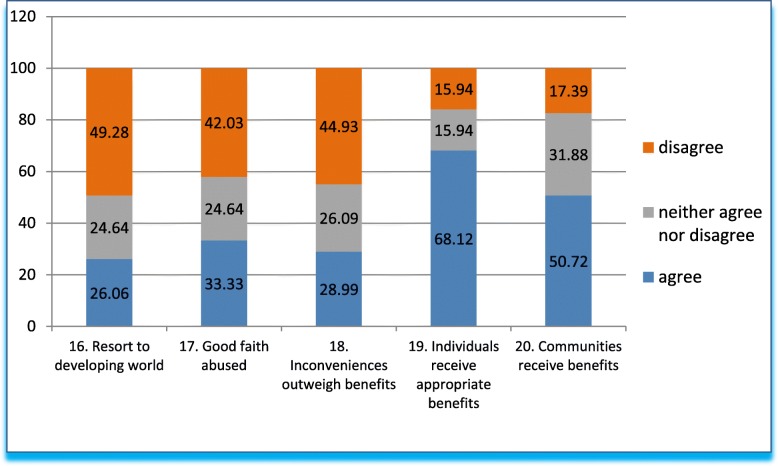
Answers to questions 16–20 in grouped percentages

Q16 to Q20 (Fig. 4) explores the issues typically associated with exploitation, i.e., due to non-optimal conditions in high income nations, pharmaceutical companies go to LMICs for the laxness in regulation and in balancing benefit/risk.

Q16 explores an external stimulus for ethics dumping, the frequently cited reason that research centers in HICs are saturated, which leads them to resort to LMICs. Only a fourth of respondents think this is so; almost half do not agree, and the rest (almost another fourth) neither affirms nor denies it.

Q17 queries whether clinical trials abuse the patients’ good faith, availability and readiness to sacrifice time and effort during enrollment. In round figures, one third of the respondents agree with the statement, 40% disagree, and a quarter neither affirms nor deny it.

Q18-Q20 explores the fairness of distribution of benefits in clinical trials. Q18 asks whether the inconveniences of taking part in a trial (traveling, appointments, wait times, other discomforts) outweigh the benefits. Like the preceding question, close to a third of respondents believe this is so, two fifths disagree, and a fourth remains neutral.

Q19 and Q20 deal concretely with Helsinki postulates, which state that trials must benefit participating individuals and their communities [12]. In the case of benefits for individuals (Q19), two thirds of respondents agree that they receive appropriate benefits, 15% disagree and 15% are neutral. Responses differ slightly when it comes to communities (Q20): half of respondents agree that communities receive benefits, a fifth disagree, while the neutral answers increase to one third.

### Norms facilitating ethics dumping

**Fig. 5:**
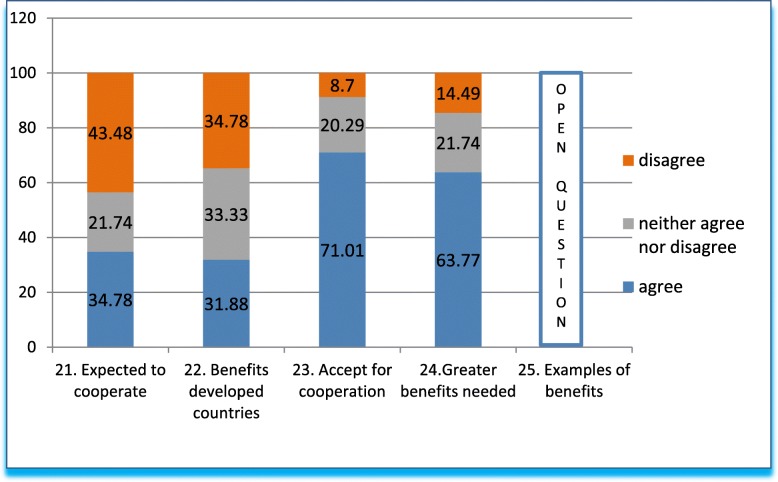
Answers to questions 21–25 in grouped percentages

Questions 21–25 (Fig. 5). Q21-Q23 explored norms that could facilitate ethics dumping. Q21 states that in HICs, the population is expected to cooperate with research altruistically, and that this applies to LMICs too. A third of respondents agree, approximately 40% disagree, and a third remain neutral. As to clinical research in general, we asked whether clinical research is conducted for the benefit of HICs first, and only secondarily for the benefit of the LMICs (Q22). Opinions were split equally: 33% for each, affirmative, negative, and neither affirmative nor negative.

Next, we analyzed respondents’ perspectives on the willingness needed to cooperate with research, stating that we should accept the current conditions of clinical research in the interest of humanitarian, altruistic and benevolent collaboration (Q23). More than two thirds of respondents agreed, less than 10% disagreed, and one fifth neither agreed nor disagreed (or were uncertain).

### Acceptable level of benefit to Mexico

In Questions 24–25 (Fig. 5, Table 3), we asked questions relating to benefit to Mexico in general due to international research. In Q24, we asked whether the price of access to our population should be higher, with greater benefits for individuals and communities. Again, two thirds agreed that the requirements for access to our population should be higher (perhaps opposing altruistic participation as mentioned in the previous question); 15% disagreed, and one fifth remained neutral. Question 25 followed up by asking how the price of access could be increased; the answers to this open-ended question are shown in Table 3.

**Table 3:** Responses to Q25 on how greater benefits to Mexico in terms of clinical trials can be achieved. Answers were classified in broad categories (in italics) and are listed in decreasing order of frequency

Broad categories	Replies
Free ongoing treatment and benefits for participants (13 mentions):	Free medication following trial conclusion (8), financial compensation for participants (2), travel and meal expenses (2), coverage for patient comorbidity (1)
Additional benefits for participants and communities (9 mentions):	Continued healthcare services. (1) Sharing the profits obtained from studied products with the community. (1) Free medical support. (1) Medical education for participants. (1) Medical attention for other population groups (different from research populations). (1) Book donations for study centers. (1) Better training, wider circulation, qualified study centers, funding. (2) Increased interaction with communities. (1)
Laws and regulations (8 mentions):	Improving laws and regulations (5). Increased supervision, surveillance (2). Economic sanctions for sponsors who commit ethics violations. (1)
Budget and management (8 mentions):	Matching research and staff payments with their first-world counterparts (6). Greater economic resources and opportunities. Subsidies and donations for hospitals. (1) Better conditions for participants and researchers. (1)
Taxation (3)	Taxes for trials. (3)

### Testing the boundaries of ethics dumping and additional thoughts

**Fig. 6:**
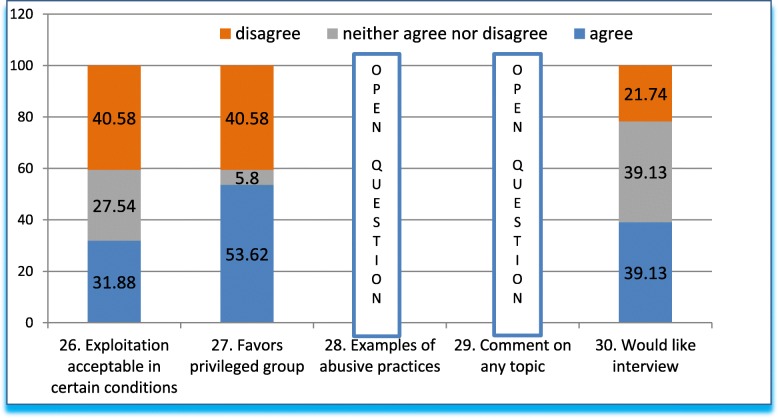
Answers to questions 26–30 in grouped percentage

Q26-Q30 (Fig. 6) tests the boundaries of the acceptability of ethics dumping by likening it to other industries. Q26 asked whether exploitation is acceptable under certain conditions. We stated that such practices are common in the automobile and textile industries, through low-cost, highly productive *maquilas* (contract manufacturing, −mass producers-, mainly for the USA), which are thought of as beneficial for our population. About one third of respondents agreed that these practices are favorable, more than a third disagreed, while other respondents (27.54%) were not sure. To explore this topic further, we asked whether the conditions of trade and economy favored a select and privileged group of mass-producers (Q27). More than half of respondents agreed, 40.58% disagreed, and only 5.8% remained neutral.

Further, Q28 sought examples of abusive practices (open question), which are summarized below (Table 4). Questions below have been grouped and classified, with the corresponding category indicated in the left column. Observations by the authors in brackets and in italics.

**Table 4:** Answers to open-ended question (Q28) on what practices could indicate abusive practices

Broad Category	Responses
Labor exploitation (11):	Long hours. Smaller budgets for developing countries than for HICs countries. Work hours. Exploitation of vulnerable (*poor*) workers. Selfish interests dominating community interests. Suspending trials prematurely when first-world quotas are met, inconveniencing patients (*ethics dumping*). Poor work and safety conditions. Work schedules. Prolonged studies. Lack of social security. Substandard work conditions (*facilities and schedules*).
Unequal wages (10 mentions):	Payment for the same work done is disproportionately lower in the LMICs.
Patient abuse (10 mentions):	Not informing patients of the benefits. Treating patients as means instead of ends. No travel or meals expenses for patients. Not giving patients enough information. Inadequate (long, unclear) informed consent. Inadequate comprehension of informed consents. Patients left unprotected when the trial ends. Utilizing patients for financial gain. Not enough time for patients to understand the informed consent. Patients not being able to afford the medication after the end of the trial. Poor economic compensation for participants.
Researchers (7 mentions):	Pressure to fulfill enrollment quotas. Researchers selected because of the volume, not the quality, of their work, compromising trial safety and the integrity of results. Inappropriate recruitment, falsifying information. Privileging the protocol over safety. Fabricating information. Researchers lacking knowledge necessary for the study. Lack of participation in publishing.
Sponsors (3 mentions):	Testing medications whose use is restricted in their country of origin (*ethics dumping*). Failure to report results. Failure to comply with regulations. Providing incomplete drug information, not disclosing adverse effects (*ethics dumping*).

Question 29 was also open, inviting respondents to contribute whatever additional comments they might have on any topic (Table 5):

**Table 5:** Summarized replies on additional comments

#	Summarized replies
1.	No [effective] participation of hospital directives in research committees
2.	There is exploitation and harassment of vulnerable groups in companies due to the pressure to recover investments
3.	Lax inclusion criteria that increase the likelihood of adverse events
4.	Medical representatives use aggressive persuasion strategies
5.	Research teams are paid less, patients receive less economic support for expenses and materials are of poorer quality than in HICs countries
6.	Better laws for research units are needed
7.	Improve regulations on post-trial information and treatment for patients
8.	Authorities abuse their power
9.	National registry of qualified researchers [to be started by the authorities]; presence of unqualified personnel; national registry of committee members
10.	It is necessary funding of the research industry (CONACYT [National Science and Technology Council] funding is insufficient), under the guidance of health professionals.
11.	Wages are lower, while the work is more difficult
12.	Academic recognition for researchers
13.	A handbook of research ethics procedures, regulations, social dimensions, etc. is necessary
14.	Create high-performance research centers to attract investment
15.	Need for quality medications at a fair price

Finally, question 30 was meant to investigate participants’ willingness to collaborate in follow-up studies and overall interest in the subject. Two fifths agreed, another two fifths neither agreed nor disagreed, and around 20% (one fifth) disagreed to the final question 30.

## Discussion

In this exploratory study, we searched the thoughts of M-REC members on the phenomenon called “ethics dumping.” Our findings demonstrated the current line of thought of M-REC members on this phenomenon, though, admittedly, further studies are necessary to factually validate their thoughts and explore the issue further. Nevertheless, since ethics committee members are the vanguards of ethics [13], having an insight into the thoughts of ethics committee members is valuable as it points to future directions on what to look at to explore the phenomenon in the Mexican and comparable contexts and search for solutions.

Our results show that though M-REC members generally evaluate clinical trials as ethical, for almost half of the respondents, ethics dumping commonly occurs and that its occurrence is something serious. For most, ethics dumping must be addressed. Most of the M-REC members were also of the opinion that there is still a lot of room for improvement when it comes to the ethical aspects of clinical trials, especially those aspects related to patient participation (e.g., improving trial conditions, patient safety, human rights, patient autonomy, informed consent, etcetera) and ethics committees (e.g., training of members, choice of members, auditing of the committee, etcetera).

Though almost most of the respondents think that ethics dumping is serious, very few could say that they could provide an example or two. We can speculate why this is so, and a possible explanation is the newness of this term and/or the lack of usage of the term in M-REC deliberations.

During the expert consultation for the questionnaire, exploitation risks related to voluntariness was a very strong theme. Indeed, when asked about “exploitation risks” related to voluntariness, our results show that the committee members were leaning towards the opinion that there are indeed voluntariness-related issues, such as undue inducement and therapeutic misconception, and that these issues may be taken advantage of by trial recruiters.

Closely related to voluntariness issues are norms that impinge on the participants’ feeling of being required to be part of a study. M-REC members believe that patients are not required to participate; however, if they do participate, they ought to accept the current conditions of clinical trials for benevolent/altruistic reasons. This closely ties with the committee members’ undecidedness whether trials are done primarily for the needs of HICs. It is possible that M-REC members would require the acceptance of the current conditions, albeit the earlier raised issues, because there is no consensus if trials are done primarily for the interests of HICs. Their undecidedness on the issue of the primary purpose of clinical trials could also impinge on the majority opinion that the price for international sponsors accessing the Mexican population should not be higher than what it is now. However, if this price ought to be increased anyway, this should foremost come in the form of free treatment and benefits for patients.

Aside from themes related to voluntariness, there were also themes related to benefit/risk evaluation. Almost a majority disagrees that the inconveniences of clinical trials outweigh the benefits, and more than the majority believe that individuals and communities receive appropriate benefits from these trials. We can speculate, though we are unable to confirm, that the judgment of general ethical acceptability of clinical trials, despite the ethical issues that need to be addressed, may be related to this balancing act. It could be that for the M-REC members, these benefits to the individuals and the community outweigh all the ethical issues we mentioned above.

Lastly, on the theme of boundaries of ethics dumping, committee members were undecided whether exploitation, in the form of low-cost productive work, may be acceptable, though the majority agreed that trade and economy favor the privileged. Low-cost productive work elicits M-REC members’ perspective on the practice of off-shoring, i.e., of bringing a business outside the sponsor’s country for some form of financial benefit. Off-shoring is an essential element of ethics dumping. Though committee members were undecided whether off-shoring is exploitation, other exploitative labor practices versus patients and local personnel were the oft-cited examples of exploitation.

This study, given its exploratory nature, is unable to answer why the responses of the M-REC members were such. It remains to be understood, for example, how M-REC members define a “generally ethical clinical trial” considering that these same members consider specific issues as serious enough to warrant some form of action. It also remains to be understood what the role of benefit-risk balancing might be in defining a trial as generally ethically compliant. Also, further research could provide some light why M-REC members are undecided on the primary purpose of international clinical trials when it was almost a majority opinion that ethics dumping commonly occurs in Mexico.

We embarked on this study to find out the thoughts of M-REC members on ethics dumping, largely to provide us with some direction on what future areas of research might be on this topic. We believe we have successfully done that, especially since we were able to identify their perspectives on specific ethics dumping-related issues, and more importantly, what about these issues need to be further understood. At the same time, this knowledge of their perspective on this issue provides a very preliminary glimpse on the capability of and extent that ethics committees protect research participants from exploitation, as well as their ability “to recognize culturally sensitive ethical issues in complex settings” [14].

### Limitations

Our findings are naturally limited by the preliminary and exploratory nature of our study, as well as by survey as a method of gathering data. This would mean that other studies should be undertaken to confirm these findings. Another limitation is related with our not so broad experience in Mexico with committees (since about 2012, where committees came into effect by law), where our knowledge so far is being gathered more recently. Comparisons with more experienced countries therefore are possibly limited.

## Conclusion

From the Mexican context and specifically from the perspective of Mexican research ethics committee members, ethics dumping commonly exists in Mexican clinical trials, as well as several related issues such as ethical issues on patient participation and ethics committees, as well as voluntariness issues such as undue inducement and therapeutic misconception. Further, most members believed these issues need to be addressed. However, most were also of the opinion that clinical trials are generally ethically compliant. This points to the need for further studies on the reasons for these perspectives.

## Additional file

Additional file 1: Appendix. Questions – Translated version (DOCX 15 kb)

## Data Availability

All data generated is stored safely by the main investigator and may be available upon request.
