# Supplementary material for: A survey in Mexico about ethics dumping in clinical research
Source: BMC Med Ethics. 2019 Jun 3;20:38. doi: 10.1186/s12910-019-0378-6 (PMC6547477; doi:10.1186/s12910-019-0378-6)
Supplement: Supplementary file 1 — Appendix. Questions – Translated version (DOCX 15 kb) [file 12910_2019_378_MOESM1_ESM.docx]

1. Are you involved in international clinical research?
2. Is research ethically conducted in our country?
3. Do you think that the ethical aspects of research should be reviewed better in our country?
4. If your answer is yes in #3, in what areas? ______________________________________
5. Have you heard the term "Ethics Dumping" (defined as the exportation of unethical practices in research that are not acceptable in developed countries)?
6. Do you think that there is ethics dumping in our country?
7. Would you describe the situation as grave and urgent?
8. Do you have an example of this type of practice at hand?
9. Would you have more than one example of these practices?
10. Should we act to stop these practices?
11. Do you feel compromised when you talk about these issues?
12. Can the offer of free medical services and transportation money influence the decision to enroll in a study?
13. Can the offer of free medical services for participants alter the results (outcomes) of a study?
14. Do participants know that the study will not bring their expected benefits to them?
15. When participants do not know that the study is not meant to bring their expected results, does this favor the recruitment of patients and provide an advantage to recruiters?
16. Do sponsors from high income countries resort to doing their researches in developing countries because high income countries are already saturated in terms of clinical trials?
17. Is the availability willingness of patients to make sacrifices in terms of time and effort abused when they are recruited for commercial studies that do not provide direct benefits?
18. Do study inconveniences (consultations, travel, waiting, discomforts) outweigh the benefits given?
19. Do companies provide the individual benefits stated in the Declaration of Helsinki?
20. Do companies provide the community benefits stated in the Declaration of Helsinki?
21. The first world takes for granted its population needs to cooperate selflessly with clinical investigation. Is this applicable to participants from developing countries?
22. To what extent is research performed to benefit the developed world and less the developing world?
23. Should we accept the current conditions in the interest of humanitarian harmony, altruistic cooperation, and goodwill?
24. Should we increase the access price to our populations of transnational research, benefiting individuals and communities in return?
25. How could this latter take effect? ________________________________________________________
26. Could exploitation be acceptable in other fields of trade (for example, in the automotive or garment industry, etc.)?
27. Do the conditions of trade and economy favor a select and privileged group of mass producers?
28. What practices can indicate abuse? _____________________________________________________
29. Would you like to comment or elaborate on any topic in more detail? ___________________________
30. I would like to participate in follow-up studies (If yes, please provide contact details at the end of this survey).
